# Retinal arteriolar tortuosity and fractal dimension are associated with long-term cardiovascular outcomes in people with type 2 diabetes

**DOI:** 10.1007/s00125-021-05499-z

**Published:** 2021-06-23

**Authors:** Emmanuel Sandoval-Garcia, Stela McLachlan, Anna H. Price, Thomas J. MacGillivray, Mark W. J. Strachan, James F. Wilson, Jackie F. Price

**Affiliations:** 1 Centre for Global Health Research, Usher Institute, University of Edinburgh, Edinburgh, UK; 2 UK Statistics Authority, Edinburgh, UK; 3 Centre for Clinical Brain Sciences, University of Edinburgh, Edinburgh, UK; 4 Metabolic Unit, Western General Hospital, Edinburgh, UK; 5 MRC Human Genetics Unit, Institute of Genetics and Molecular Medicine, University of Edinburgh, Edinburgh, UK

**Keywords:** Biomarkers, Cardiovascular disease, Epidemiology, Retinal imaging, Stroke, Type 2 diabetes

## Abstract

**Aims/hypothesis:**

Our aim was to determine whether quantitative retinal traits in people with type 2 diabetes are independently associated with incident major cardiovascular events including CHD and stroke.

**Methods:**

A total of 1066 men and women with type 2 diabetes, aged 65–74 years, were followed up over 8 years in the population-based Edinburgh Type 2 Diabetes Study. Using retinal photographs taken at baseline and specialist software, a number of quantitative retinal traits were measured, including arteriolar and venular widths and tortuosity as well as fractal dimension (a measure of the branching pattern complexity of the retinal vasculature network). Incident CHD events occurring during follow-up included fatal and non-fatal myocardial infarction, first episodes of angina and coronary interventions for CHD. Incident cerebrovascular events included fatal and non-fatal stroke or transient ischaemic attack. Cox proportional hazard regression analyses were performed to identify the association of the retinal traits with cardiovascular events in the population with retinal data available (*n* = 1028).

**Results:**

A total of 200 participants had an incident cardiovascular event (139 CHD and 61 cerebrovascular events). Following adjustment for age and sex, arteriolar tortuosity and fractal dimension were associated with cerebrovascular events (HR 1.27 [95% CI 1.02, 1.58] and HR 0.74 [95% CI 0.57, 0.95], respectively), including with stroke alone (HR 1.30 [95% CI 1.01, 1.66] and HR 0.73 [95% CI 0.56, 0.97], respectively). These associations persisted after further adjustment for established cardiovascular risk factors (HR 1.26 [95% CI 1.01, 1.58] and HR 0.73 [95% CI 0.56, 0.94], respectively). Associations generally reduced in strength after a final adjustment for the presence of diabetic retinopathy, but the association of fractal dimension with incident cerebrovascular events and stroke retained statistical significance (HR 0.73 [95% CI 0.57, 0.95] and HR 0.72 [95% CI 0.54, 0.97], respectively). Associations of retinal traits with CHD were generally weak and showed no evidence of statistical significance.

**Conclusions/interpretation:**

Arteriolar tortuosity and fractal dimension were associated with incident cerebrovascular events, independent of a wide range of traditional cardiovascular risk factors including diabetic retinopathy. These findings suggest potential for measurements of early retinal vasculature change to aid in the identification of people with type 2 diabetes who are at increased risk from stroke.

**Graphical abstract:**

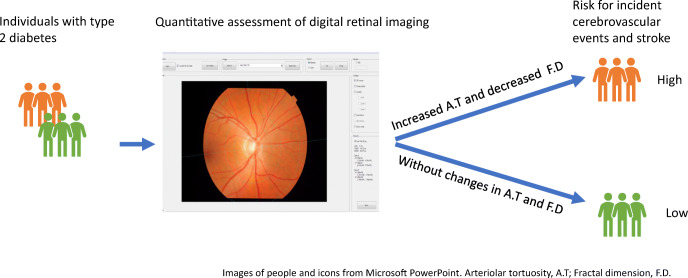

**Supplementary Information:**

The online version contains peer-reviewed but unedited supplementary material available at 10.1007/s00125-021-05499-z.

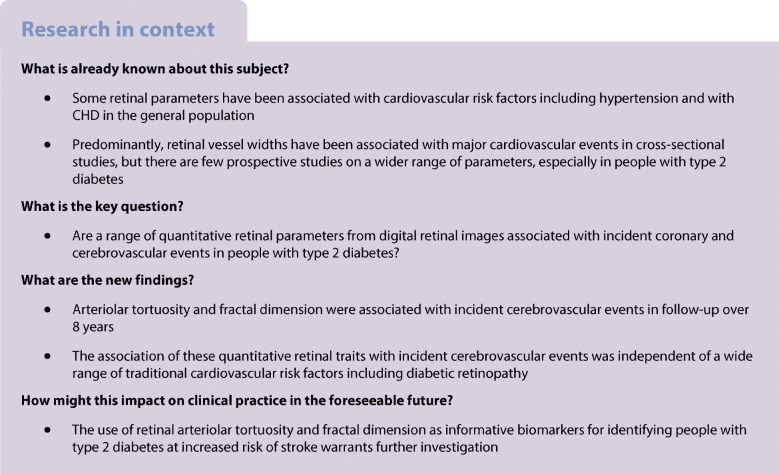



## Introduction

People with type 2 diabetes have a well-established increased risk of CVD, including CHD, peripheral arterial disease and cerebrovascular disease [1, 2]. Despite the substantial interest in evaluating cardiovascular risk at an individual level, there is no general consensus on which biomarkers are most useful for predicting the development of CVD in this population. Current ‘traditional’ biomarkers and CVD risk prediction models in people with type 2 diabetes lack accuracy [3], indicating a need for novel biomarkers that predict individual risk in this population.

Recent studies indicate the potential of novel retinal biomarkers, related to measurable morphology of the retinal vasculature observable in fundus camera images and analysed by computer-based platforms, to assess risk of major cardiovascular events in type 2 diabetes [4, 5]. With advances in technology, retinal imaging is reproducible, radiation free, and relatively inexpensive. Changes in the retinal microvasculature are associated with vascular risk factors and microvascular complications in healthy individuals and in individuals with type 2 diabetes, such as hypertension and diabetic retinopathy [5, 6]. These changes in retinal microcirculation have also been associated with the presence of major atherosclerotic cardiovascular outcomes, including CHD [7] and stroke [8, 9]. However, the majority of studies to date have been cross-sectional, limiting the findings in relation to the assessment of the temporal relationship between biomarkers and subsequent onset of cardiovascular events. Moreover, research in this area, specifically in people with type 2 diabetes, is extremely limited.

A wide range of retinal vessel traits have been investigated [10], including measurements of arteriolar and venular diameters; branching complexity of the vascular network or pattern quantified through the calculation of fractal dimension; and tortuosity which puts a numerical value to how much a vessel twists and turns.

In light of the potential for retinal vascular traits to predict development of cardiovascular events, and the current lack of prospective studies, particularly in people with type 2 diabetes, the aim of the current study was to determine the association between quantitative retinal traits and subsequent development of major cardiovascular events in people with type 2 diabetes.

## Methods

### Study design

The Edinburgh Type 2 Diabetes Study (ET2DS) is a population-based, prospective cohort of 1066 men and women aged between 60 and 75 years at baseline (2006–2007) with an established diagnosis of type 2 diabetes, living in the Lothian region of Scotland, UK. Details of the study protocol have been published previously [11]. Participants were randomly selected by sex and 5 year age bands from the Lothian diabetes register, a comprehensive database of people with type 2 diabetes living in Lothian. Of the 5454 individuals who were invited to participate, 1066 were recruited to the study and were shown subsequently to be largely representative of the target population of all older men and women with type 2 diabetes in Lothian [12]. Participants attended baseline research clinics for physical examination and retinal photography, and were subsequently followed up at 4 and 8 years for cardiovascular events. The ET2DS was granted ethical approval by the Lothian Medical Research Ethics Committee, and all participants gave written informed consent.

### Baseline physical examination

At the main baseline ET2DS clinic, demographic, medical history and clinical variables were collected. A self-completion medical questionnaire was completed and brachial BP was measured. Fasting venous blood was obtained for analysis of fasting glucose, total cholesterol, HDL-cholesterol, HbA_1c_ and serum creatinine. Total cholesterol, HDL-cholesterol, HbA_1c_ and creatinine were all measured using Vitros Fusion Chemistry System (Ortho Clinical Diagnostics, UK) at the Western General Hospital, Edinburgh, UK. Plasma C-reactive protein (CRP) was measured at the University Department of Medicine, Glasgow Royal Infirmary, UK, using a high-sensitivity immunonephelometric assay. eGFR was calculated from creatinine results using the Chronic Kidney Disease Epidemiology Collaboration (CKD-EPI) equation.

### Retinal photography and retinopathy grading

Retinal images were collected at baseline in the main ophthalmology department for NHS Lothian by a single specially trained medical photographer. Of the 1046 ET2DS participants who underwent retinal examinations [13], two were subsequently excluded from analyses as they did not have gradable photographs, leaving 1044 participants. During the assessment, participants had 1% tropicamide drops instilled into both eyes to allow pupillary dilatation before having the image captured. If, on inspection of the pupils, dilatation was insufficient, a further 1% tropicamide dose was given. Retinal images were taken at 45° angle using a digital fundus camera (TOPCON TRC-NW8, Topcon Optical Company, Tokyo, Japan). Standard 7-field non-stereoscopic retinal colour photographs were taken, and assessed for the presence of diabetic retinopathy. Two trained optometrists graded all the photographs, working independently and according to the scale described by the Early Treatment Diabetic Retinopathy Study research group [14, 15]. For each eye, the maximum grade in any of the seven photographic fields was determined for each of the characteristic lesions of diabetic retinopathy and was used in defining the final retinopathy levels, varying from level 10 (no retinopathy) to level 81 (advanced proliferative retinopathy). Any discrepancies between the scores assigned by the two graders were resolved through discussion between the graders with any unresolved discrepancies being reviewed and arbitrated by an ophthalmologist. Diabetic retinopathy was classified as a binary variable for the purpose of the current analysis. Any degree of diabetic retinopathy (e.g. mild, moderate and/or severe) were included as cases.

### Measurement of retinal quantitative traits

**Fig. 1:**
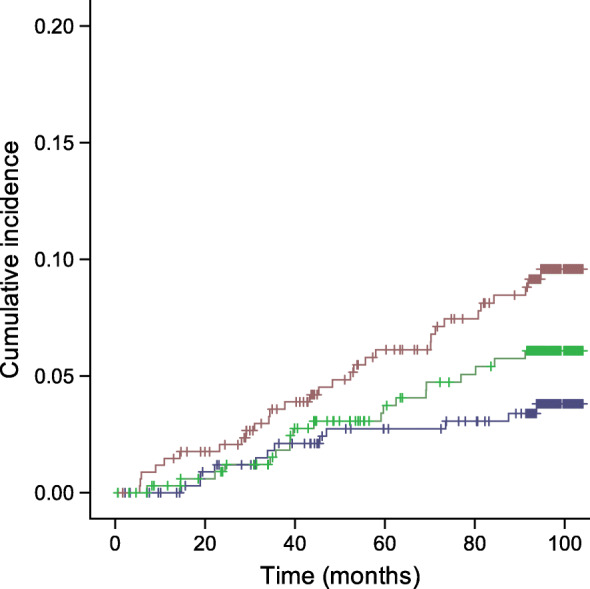
Kaplan–Meier curve demonstrating cumulative incidence of cerebrovascular events stratified according to baseline arteriolar tortuosity tertiles (log_e_ transformed). Blue line, tertile 1; green line, tertile 2; brown line, tertile 3

For each participant, the best quality central image (either right or left side) was selected to be included in the analysis of quantitative retinal traits. Vascular Assessment and Measurement Platform for Images of the Retina (VAMPIRE) version 3.1 software [16] was used to quantify retinal vessel traits including the widths of the six longest arterioles to calculate the central retinal arteriolar equivalent (CRAE); the six longest venules to calculate the central retinal venous equivalent (CRVE); and the ratio of these two parameters to give the arteriovenous ratio (AVR). These parameters are located in zone B. Measurement of these vessel widths is based on cross-sectional width at regular intervals along the length of a vessel segment (electronic supplementary material [ESM] Fig. 1).

Arteriolar tortuosity (mean measurement from the six thickest arterioles in a single participant) and venular tortuosity (mean measurement from the six thickest venules in a single participant) were taken from zone C. This is a ring-shaped area located between 0.5 and 1 disc diameter’s distance from the optic disc. The analyses of global vasculature (arterioles and venules) extracted the multifractal dimension, which is an extension of fractals in two-dimensional space using the sand-box method and has been described elsewhere [17]. For the purpose of this study, multifractal dimension will be referred to as fractal dimension. Obtaining fractal dimensions involved automatic segmentation classifying each pixel of the retinal image as vessel and non-vessel to produce a segmented map of the retinal vasculature. In a step prior to fractal analysis, computationally segmented images were inspected and artefacts (e.g. low contrast) caused by dust on the camera were corrected or removed. This iterative deletion of the pixels produced skeletonised images which are required to obtain fractal dimension. These analyses were performed after processing and stored the initial results.

To evaluate reliability and agreement of the measured retinal traits, interclass correlation coefficients were computed to measure intra- and inter-grader agreement [18]. Depending on the precise retinal area from which readings are taken, intra-grader and inter-grader correlations for these retinal traits using VAMPIRE have been shown to be high to very high previously [19].

### Identification of CVD

#### Prevalent CVD at baseline

The presence of CVD at baseline was determined using a combination of self-report in the standardised ET2DS self-completion questionnaire, the WHO chest pain questionnaire, a 12-lead ECG coded using the Minnesota coding system and linkage to hospital discharge data from the information service division of NHS Scotland (ISD). Presence of CVD included myocardial infarction, angina, stroke, transient ischaemic attack (TIA) and/or coronary intervention defined according to previously reported criteria which were based on these sources of information [11]. Additionally, when necessary, clinical notes from general practices and hospitals were obtained to confirm or refute a possible diagnosis of CVD. The presence of prevalent CVD was included as binary variable, categorising any type of event as cases.

#### Incident cardiovascular events during follow-up

Incident and recurrent cardiovascular events were recorded at the 4 year follow-up phase of the ET2DS and after 8 years, based on the same predefined criteria as at baseline. At year 4, events were identified using self-completion questionnaires completed in the 4 year research clinic, questionnaires from general practitioners (GPs) (including from clinic non-attenders), ECGs and record linkage to hospital discharge data from ISD. If necessary, clinical notes from the hospital were searched to confirm clinical information. After 8 years from baseline, repeat record linkage was undertaken to identify further cardiovascular events.

Specific criteria used to define an incident cardiovascular event (fatal and non-fatal) were as follows. Myocardial infarction: ICD-10 (International Classification of Diseases, 10th revision) code for new myocardial infarction (I21-I23, I25.2) on discharge/death record, dated after baseline, confirmed by self-reported doctor diagnosis of myocardial infarction; positive WHO chest pain questionnaire for myocardial infarction; report of myocardial infarction on GP questionnaire; new ECG changes; or inspection of clinical notes. Angina: (1) ICD-10 code for angina (I20, I25) as primary diagnosis on hospital discharge record, dated after baseline, with no previous indication of angina; or (2) at least two of (a) self-reported doctor diagnosis of angina or new angina medication since baseline, (b) ECG codes for ischaemia that were not present at baseline and (c) positive WHO chest pain questionnaire. Fatal CHD: ICD-10 code for CHD (I209, I249, I258, I259) as primary cause of death. Stroke: (1) ICD-10 code for stroke (I61, I63, I66, I679, I694) as primary diagnosis on discharge/death record, dated after baseline, or (2) self-report of stroke or non-primary ICD-10 discharge/death code for stroke dated after baseline, both confirmed on scrutiny of clinical notes. TIA: (1) ICD-10 code for TIA (G45) as primary diagnosis on discharge record; or (2) self-report of stroke or non-primary ICD-10 discharge code for stroke or TIA dated after baseline, confirmed as TIA on scrutiny of clinical notes. Coronary intervention: OPCS operation code for coronary intervention (K40-K44) on discharge record.

### Statistical analyses

Initially, all variables were examined for outliers/extreme values and missing values prior to undertaking any statistical analyses. All the continuous variables were assessed visually for normality using histograms in an effort to address potential bias. Those variables that were not normally distributed were transformed using logarithm base e (log_e_). Rank transformation was used to normalise the distribution of fractal dimension, because fractal dimension is a unitless feature of structural complexity. In regression analyses, different units and different scales are often used. Given that this may cause difficulty in interpreting the results and comparison across findings, values were standardised using the mean and SD to produce a scaled variable. This maintains the same relationship to one another as the unscaled variable. Univariable analyses of established cardiovascular risk factors and quantitative retinal traits were run to detect differences between those with incident cardiovascular event during follow-up and those without incident cardiovascular events. The *χ*^2^ test was used to explore relationships between dichotomous variables. The unpaired *t* test or Mann–Whitney *U* test were applied to assess the differences of continuous variables in people with incident and without incident cardiovascular events. Results are expressed as means (± SD) unless otherwise stated.

#### Prospective statistical analysis

Cox proportional hazard survival regression analyses were used to examine the association of the baseline quantitative retinal traits with incident cardiovascular events. Variables were selected as possible confounders to build different models and assess the strength and independence of the association of the quantitative retinal traits with incidental cardiovascular outcomes. Covariables were selected according to the significant univariate association results and also to reflect traditional risk factors described previously in the literature. HRs for continuous variables relate to a 1 SD increase. False discovery rates were calculated to understand the rate of type 1 errors when carrying out multiple tests. Event-free survival curves for cerebrovascular events were developed using the Kaplan–Meier method. Difference of incidence between groups was assessed by logrank tests.

Quantitative retinal traits found to be independently associated with incident events were evaluated, in combination with traditional cardiovascular risk factors, using concordance statistic (or C statistic) to see if the model improved, to evaluate the discriminative ability of the models, before and after addition of the retinal trait. Calibration was assessed using an extension of the Hosmer–Lemeshow χ^2^ statistic to test goodness-of-fit (*p* value >0.05 indicates good calibration). Statistical analyses were carried out using SPSS (version 23.0; IBM, Armonk, NY, USA) and R (R: A language and environment for statistical computing, version 3.1.1, Vienna, Austria). A *p* value of less than 0.05 was taken as statistically significant.

## Results

The baseline demographic, vascular disease and retinal trait characteristics of the ET2DS population are summarised in Table 1. A total of 547 (51.31%) participants were male and the mean age was 67.9 years at baseline. Prevalence of prior myocardial infarction, angina, stroke, TIA and coronary intervention were 150 (14.1%), 298 (27.5%), 62 (5.8%), 31 (2.9%) and 110 (10.3%), respectively. The prevalence of diabetic retinopathy at baseline was 31.8%.

**Table 1:** Baseline characteristics of the ET2DS population (maximum *N* = 1066)

Variable	
Age (years)	67.9 ± 4.2
Sex (male)	547 (51.3)
BMI (kg/m^2^)	31.4 ± 5.7
BP	
Systolic (mmHg)	133.3 ± 16.4
Diastolic (mmHg)	69.1 ± 9.0
Plasma glucose (mmol/l)	7.5 ± 2.1
Total cholesterol (mmol/l)	4.31 ± 0.9
HbA_1c_	
(mmol/mol)	57 ± 8.6
(%)	7.4 ± 1.1
HDL-cholesterol (mmol/l)	1.3 ± 0.4
eGFR (ml min^−1^ [1.73 m]^−2^)	78.3 ± 23.1
CRP (mg/l)	1.9 (0.9–4.4)
Duration of diabetes (years)	6.0 ± 8.0
Current diabetes treatment	
Diet only	200 (18.8)
Tablets	675 (63.4)
Insulin ± tablets	190 (17.8)
Anti-hypertensive medication	872 (81.8)
Lipid-lowering medication	912 (85.6)
Smoking	
Current smoker	153 (14.4)
Ex-smoker	499 (46.8)
Never smoker	414 (38.8)
CV event^a^	
Myocardial infarction	150 (14.1)
Angina	298 (27.5)
Stroke	62 (5.8)
TIA	31 (2.9)
Coronary intervention	110 (10.3)
Diabetic retinopathy	
Absent	705 (66.1)
Present	339 (31.8)
Quantitative retinal traits	
AVR	0.7 ± 0.9
CRAE (px)	33.1 ± 3.9
CRVE (px)	45.0 ± 5.0
Arteriolar tortuosity	3.9 (0.13–279.0)^b^
Venular tortuosity	4.6 (0.26–410.0)^b^
Fractal dimension	1.8 (1.5–1.9)

Data are presented as mean ± SD, median (IQR), or *n* (%)

^a^Note that there is overlap between these event subgroups

^b^Values 10^−5^

CV, cardiovascular; px, pixels

In the total population with retinal data available (*n* = 1028) and after a mean follow-up of 7.2 years (range 0.05–8.5, median 7.8 years), the number of first incident cardiovascular events was 200 (19%), including 60 fatal/non-fatal myocardial infarction, 36 angina, 49 stroke, 12 TIA, 26 coronary intervention and 17 fatal CHD.

A comparison of baseline variables according to whether or not participants developed an incident cardiovascular event is shown in Table 2. As expected, participants with incident cardiovascular events were more likely to be male, were older, had lower eGFR and HDL-cholesterol levels, longer diabetes duration, higher HbA_1c_, and were more likely to have prevalent cardiovascular disease and diabetic retinopathy. Participants on anti-hypertensive medication and those using medication for glucose control were more likely to develop incident cardiovascular events.

**Table 2:** Baseline characteristics of individuals with incident CV events and no events (*n* = 1028 with retinal trait data)

Variable	Incident event (maximum *n* = 200)	No incident event (maximum *n* = 828)	*p* value
Age (years)	68.9 ± 4.2	67.7 ± 4.2	<0.001
Sex (male)	118 (59.0)	410 (49.5)	<0.01
BMI (kg/m^2^)	31.9 ± 5.6	31.3 ± 5.7	0.23
BP			
Systolic (mmHg)	134.9 ± 19.1	132.8 ± 15.6	0.10
Diastolic (mmHg)	69.1 ± 9.8	69.0 ± 8.8	0.92
Plasma glucose (mmol/l)	7.8 ± 2.6	7.5 ± 1.9	0.13
Total cholesterol (mmol/l)	4.4 ± 1.0	4.3 ± 0.9	0.06
HbA_1c_			
(mmol/mol)	60 ± 11	56 ± 8.6	<0.01
(%)	7.6 ± 1.3	7.3 ± 1.0	<0.01
HDL-cholesterol (mmol/l)	1.2 ± 0.3	1.3 ± 0.4	<0.0001
eGFR (ml min^−1^ [1.73 m]^−2^)	70.4 ± 23.9	80.0 ± 22.6	<0.0001
CRP (mg/l)	4.0 (1.2–4.6)	3.9 (0.8–4.4)	0.76
Duration of diabetes (years)	9.3 ± 6.8	7.8 ± 6.3	<0.01
Current diabetes treatment ^a^			
Oral diabetes medication	142 (71.0)	607 (73.3)	0.49
Insulin ± tablets	57 (28.5)	128 (15.4)	<0.0001
Anti-hypertensive medication	26 (13.0)	154 (18.6)	<0.05
Lipid-lowering medication	21 (10.5)	122 (14.7)	0.75
Smoking			
Current smoker	28 (14.0)	118 (14.2)	0.78
Ex-smoker	100 (50)	385 (46.5)	0.35
Never smoker	72 (36.0)	325 (39.3)	0.64
CVD at baseline	116 (58.0)	248 (30.0)	<0.0001
Diabetic retinopathy			
None	118 (59.0)	587 (70.9)	<0.01
Present	86 (43.0)	253 (30.6)	<0.01
Quantitative retinal traits			
AVR	0.74 ± 0.7	0.74 ± 0.8	0.34
CRAE	32.67 ± 3.7	33.11 ± 3.9	0.14
CRVE	44.46 ± 4.9	44.72 ± 5.0	0.50
Arteriolar tortuosity	−10.01 ± 1.2	−10.09 ± 1.1	0.40
Venular tortuosity	−9.92 ± 1.0	−9.97 ± 0.9	0.55
Fractal dimension	1.7 ± 0.1	1.8 ± 0.1	0.08

Data are presented as mean ± SD, median (IQR), or *n* (%)

Values for arteriolar and venular tortuosity were normalised using log_e_ transformation

Rank transformation was used to normalise the data of the fractal dimension variable

^a^Note there were 93 (11.2%) participants on diet only with no incident event

CV, cardiovascular

Although there were no statistically significant differences between the groups (e.g. non-incident cardiovascular and incident cardiovascular), the direction of non-significant differences can be seen, i.e. participants with incident events tended to have narrower arterioles (as indicated by a decreased CRAE), narrower venules (decreased CRVE), increased arteriolar tortuosity, increased venular tortuosity and decreased fractal dimension (indicating a sparser and less complex vasculature). These details are described in Table 2.

Multivariable Cox regression model findings are shown in Table 3 for vessel widths, and Table 4 for arteriolar tortuosity and fractal dimension. Significant models using tertiles of arteriolar tortuosity and tertiles for fractal dimension are shown in Tables 5 and 6, respectively.

**Table 3:** Association of AVR, CRAE and CRVE with incident CV events in Cox regression analyses

Event	AVR			CRAE			CRVE		
	HR	95% CI	*p* value	HR	95% CI	*p* value	HR	95% CI	*p* value
Any CV event (*n* = 200)									
Model A	0.39	0.07, 2.32	0.30	0.97	0.94, 1.01	0.15	0.99	0.96, 1.01	0.56
Model B	0.38	0.07, 2.22	0.29	0.98	0.94, 1.01	0.34	0.99	0.97, 1.02	0.92
Model C	0.49	0.09, 2.83	0.43	0.97	0.93, 1.00	0.09	0.98	0.96, 1.01	0.24
Coronary events (*n* = 139)									
Model A	0.42	0.05, 3.59	0.43	0.97	0.93, 1.02	0.32	0.99	0.96, 1.02	0.68
Model B	0.41	0.05, 3.33	0.41	0.98	0.94, 1.03	0.53	1.00	0.96, 1.03	0.99
Model C	0.56	0.07, 4.58	0.59	0.97	0.93, 1.02	0.19	0.98	0.94, 1.01	0.28
Cerebrovascular events (*n* = 61)									
Model A	0.32	0.01, 8.19	0.49	0.96	0.90, 1.03	0.28	0.98	0.94, 1.03	0.67
Model B	0.33	0.01, 8.11	0.50	0.97	0.91, 1.04	0.42	0.99	0.94, 1.04	0.88
Model C	0.34	0.01, 8.23	0.52	0.96	0.90, 1.03	0.49	0.99	0.94, 1.04	0.66

Model A: unadjusted. Model B: basic model adjusted for sex and age. Model C (multivariate, fully adjusted for CV risk factors): adjusted for sex, age, duration of diabetes, systolic BP, HbA_1c,_ total cholesterol, HDL-cholesterol, smoking, prevalent baseline CVD, eGFR and diabetic retinopathy

CV, cardiovascular

**Table 4:** Association of arteriolar tortuosity and fractal dimension traits with incident CV events in Cox regression analyses

Event	Arteriolar tortuosity			Fractal dimension		
	HR	95% CI	*p* value	HR	95% CI	*p* value
Any CV event (*n* = 200)						
Model A	1.05	0.93, 1.18	0.42	0.89	0.77, 1.02	0.10
Model B	1.05	0.93, 1.19	0.39	0.90	0.78, 1.03	0.15
Model C	1.05	0.92, 1.19	0.44	0.91	0.79, 1.05	0.24
Model D	1.04	0.92, 1.18	0.55	0.89	0.77, 1.02	0.11
Coronary events (*n* = 139)						
Model A	0.97	0.83, 1.12	0.67	0.97	0.82, 1.14	0.73
Model B	0.97	0.84, 1.12	0.71	0.98	0.83, 1.16	0.87
Model C	0.96	0.82, 1.12	0.61	0.99	0.83, 1.18	0.98
Model D	0.95	0.82, 1.11	0.54	0.97	0.81, 1.16	0.75
Cerebrovascular events (*n* = 61)						
Model A	1.26	1.02, 1.57	0.03	0.73	0.57, 0.94	0.01
Model B	1.27	1.02, 1.58	0.03	0.74	0.57, 0.95	0.02
Model C	1.26	1.01, 1.58	0.04	0.73	0.56, 0.94	0.02
Model D	1.26	1.01, 1.58	0.04	0.73	0.57, 0.95	0.01
Model E	1.24	0.98, 1.57	0.07	0.71	0.54, 0.92	0.01
Stroke events (*n* = 49)						
Model A	1.29	1.01, 1.65	0.04	0.72	0.55, 0.96	0.02
Model B	1.30	1.01, 1.66	0.03	0.73	0.56, 0.97	0.03
Model C	1.31	1.01, 1.69	0.04	0.73	0.54, 0.96	0.03
Model D	1.31	1.01, 1.68	0.04	0.72	0.54, 0.97	0.02
Model E	1.28	0.98, 1.68	0.07	0.70	0.51, 0.94	0.02

Tortuosity variable is presented and was log_e_ transformed. Fractal dimension has been rank transformed. Model A: unadjusted. Model B: adjusted for sex and age. Model C: adjusted for sex, age, duration of diabetes, systolic BP, HbA_1c_, total cholesterol, HDL-cholesterol, smoking, prevalent baseline CVD, eGFR. Model D (multivariate, fully adjusted for CV risk factors): Model C plus diabetic retinopathy. Model E: Model D plus CRP

CV, cardiovascular

**Table 5:** Association of arteriolar tortuosity with incident cerebrovascular and stroke events in Cox regression analyses with continuous and tertiles variables

		Overall HRs				
Event	Level (tertiles)	Model A	Model B	Model C	Model D	Model E
	Arteriolar tortuosity	HR (95% CI; *p* value)	HR (95% CI; *p* value)	HR (95% CI; *p* value)	HR (95% CI; *p* value)	HR (95% CI; *p* value)
Cerebrovascular events (*n* = 61)						
As continuous variables		1.26 (1.02, 1.57; 0.03)	1.27 (1.02, 1.58; 0.03)	1.26 (1.01, 1.58; 0.04)	1.26 (1.01, 1.58; 0.04)	1.24 (0.98, 1.57; 0.07)
As categorical variable	1 (*n* = 344)	1.0 (reference)	1.0 (reference)	1.0 (reference)	1.0 (reference)	1.0 (reference)
	2 (*n* = 342)	1.62 (0.79, 3.34; 0.18)	1.62 (0.78, 3.33;0.19)	1.63 (0.79, 3.38; 0.18)	1.61 (0.77, 3.35; 0.20)	1.66 (0.78, 3.56; 0.18)
	3 (*n* = 342)	2.58 (1.32, 5.05; 0.005)	2.58 (1.32, 5.07; 0.006)	2.50 (1.26, 4.95; 0.009)	2.48 (1.25, 4.93; 0.01)	2.48 (1.21, 5.07; 0.01)
Stroke events (*n* = 49)						
As continuous variables		1.29 (1.01, 1.65; 0.04)	1.30 (1.01, 1.66; 0.03)	1.31(1.01, 1.69; 0.04)	1.31 (1.01, 1.68; 0.04)	1.28 (0.98, 1.68; 0.07)
As categorical variable	1 (*n* = 344)	1.0 (reference)	1.0 (reference)	1.0 (reference)	1.0 (reference)	1.0 (reference)
	2 (*n* = 342)	1.44 (0.63, 3.23; 0.38)	1.45 (0.64, 3.26; 0.37)	1.47 (0.65, 3.33; 0.35)	1.45 (0.64, 3.30; 0.37)	1.50 (0.64, 3.55; 0.35)
	3 (*n* = 342)	2.60 (1.24, 5.40; 0.01)	2.63 (1.26, 5.52; 0.01)	2.54 (1.20, 5.39; 0.01)	2.52 (1.19, 5.36; 0.02)	2.53 (1.15, 5.60; 0.02)

Tortuosity variable is presented and was log_e_ transformed. Model A: unadjusted. Model B: adjusted for sex and age. Model C: adjusted for sex, age, duration of diabetes, systolic BP, HbA_1c_, total cholesterol, HDL-cholesterol, smoking, prevalent baseline CVD, eGFR. Model D (multivariate, fully adjusted for CV risk factors): Model C plus diabetic retinopathy. Model E: Model D plus CRP

CV, cardiovascular

**Table 6:** Association of fractal dimension with incident cerebrovascular and stroke events in Cox regression analyses with continuous and tertiles variables

		Overall HRs				
Event	Level (tertiles)	Model A	Model B	Model C	Model D	Model E
	Fractal dimension	HR (95% CI; *p* value)	HR (95% CI; *p* value)	HR (95% CI; *p* value)	HR (95% CI; *p* value)	HR (95% CI; *p* value)
Cerebrovascular events (*n* = 61)						
As continuous variables		0.73 (0.57, 0.94; 0.01)	0.74 (0.57, 0.95; 0.02)	0.73 (0.56, 0.94; 0.02)	0.73 (0.57, 0.95; 0.01)	0.71 (0.54, 0.92; 0.01)
As categorical variable	1 (*n* = 338)	1.0 (reference)	1.0 (reference)	1.0 (reference)	1.0 (reference)	1.0 (reference)
	2 (*n* = 339)	0.82 (0.47, 1.44; 0.50)	0.83 (0.47, 1.46; 0.52)	0.78 (0.44, 1.38; 0.39)	0.77 (0.43, 1.38; 0.38)	0.78 (0.43, 1.40; 0.40)
	3 (*n* = 338)	0.43 (0.21–0.85; 0.02)	0.45 (0.22, 0.88; 0.02)	0.42 (0.21, 0.84; 0.02)	0.43 (0.21, 0.85; 0.02)	0.38 (0.18–0.79; 0.01)
Stroke events (*n* = 49)						
As continuous variables		0.72 (0.55, 0.96; 0.02)	0.73 (0.56, 0.97; 0.03)	0.73 (0.54, 0.96; 0.03)	0.72 (0.54, 0.97; 0.02)	0.70 (0.51, 0.94; 0.02)
As categorical variable	1 (*n* = 338)	1.0 (reference)	1.0 (reference)	1.0 (reference)	1.0 (reference)	1.0 (reference)
	2 (*n* = 339)	0.82 (0.44, 1.54; 0.55)	0.83 (0.44, 1.55; 0.55)	0.80 (0.42, 1.51; 0.49)	0.79 (0.42, 1.51; 0.48)	0.80 (0.41, 1.53; 0.49)
	3 (*n* = 338)	0.40 (0.18, 0.82; 0.02)	0.40 (0.18, 0.88; 0.02)	0.38 (0.17, 0.84; 0.02)	0.38 (0.17, 0.84; 0.01)	0.32 (0.13, 0.76; 0.01)

Tortuosity variable is presented and was log_e_ transformed. Fractal dimension has been rank transformed. Model A: unadjusted. Model B: adjusted for sex and age. Model C: adjusted for sex, age, duration of diabetes, systolic BP, HbA_1c_, total cholesterol, HDL-cholesterol, smoking, prevalent baseline CVD, eGFR. Model D (multivariate, fully adjusted for CV risk factors): Model C plus diabetic retinopathy. Model E: Model D plus CRP

CV, cardiovascular

No evidence of statistically significant associations of composite cardiovascular, coronary or cerebrovascular events with changes in vessel widths were found (i.e. AVR, CRAE or CRVE) (Table 3). Supplemental analyses explored the association of quantitative retinal traits with composite severe cardiovascular events only (myocardial infarction, coronary intervention, fatal ischaemic heart disease and stroke). There was a sex interaction for AVR (*p* = 0.007), CRAE (*p* = 0.008), CRVE (*p* = 0.006), also seen for arteriolar tortuosity (*p* = 0.007), venular tortuosity (*p* = 0.006) and fractal dimension (*p* = 0.005). Findings of multivariate analyses using this smaller group of composite severe cardiovascular events were not statistically significant (ESM Table 1).

There were no statistically significant associations for arteriolar or venular tortuosity measures with coronary events and no association between venular tortuosity and cerebrovascular events (including stroke). However, increased arteriolar tortuosity was associated with incident cerebrovascular events (Table 4); analyses between sex and retinal traits and sex, age and retinal traits were evaluated for any effect modification. The sex interaction was statistically non-significant for cerebrovascular events. Estimated HR for sex was slightly increased in men compared with women. In unadjusted models, for 1 SD increase in the arteriolar tortuosity there was a 26% risk increase in cerebrovascular events, remaining significant after adjustment for sex, age, duration of diabetes, systolic BP, HbA_1c_, total cholesterol, HDL-cholesterol, smoking, prevalent cardiovascular events and renal function. The association was attenuated in the fully adjusted model including diabetic retinopathy and CRP.

Fractal dimension also showed statistically significant associations with cerebrovascular events and stroke (Table 4). In the unadjusted models, for a 1 SD increase in the fractal dimension, there was a decrease in risk of cerebrovascular events by 73% and stroke by 72%. These associations remained significant after full multivariable adjustment, including for diabetic retinopathy and CRP.

### Prediction analyses for arteriolar tortuosity and fractal dimension

Arteriolar tortuosity and fractal dimension were added, separately, to a base model with traditional vascular risk factors for cerebrovascular events. Regarding cerebrovascular events, for arteriolar tortuosity, the discriminative ability (C statistic) of the resultant model improved modestly from 0.705 to 0.723. For fractal dimension, the C statistic correspondingly increased from 0.705 to 0.725. For diabetic retinopathy, the C statistic resultant model improved minimally from 0.705 to 0.706. Regarding stroke, findings were similar for both the addition of arteriolar tortuosity to the base model (C statistic improved modestly from 0.706 to 0.723) and for the addition of fractal dimension (C statistic increase from 0.706 to 0.727). Corresponding goodness-of-fit analysis for the base model including vascular risk factors for cerebrovascular events showed good calibration (*p* = 0.12). Likewise, models including arteriolar tortuosity (*p* = 0.53) and fractal dimension (*p* = 0.59) were well calibrated. Findings were similar for stroke models, with good calibration found for the base model (*p* = 0.58) and those with arteriolar tortuosity (*p* = 0.26) and fractal dimension (*p* = 0.93).

**Fig. 2:**
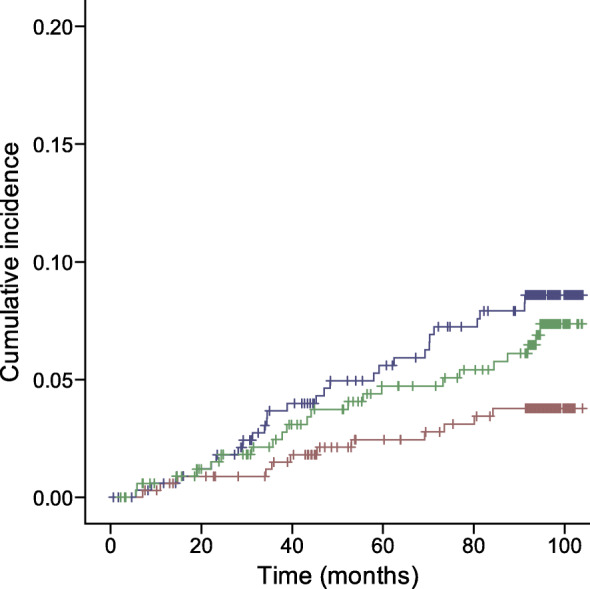
Kaplan–Meier curve demonstrating cumulative incidence of cerebrovascular events stratified according to baseline fractal dimension tertiles (rank transformed). Blue line, tertile 1; green line, tertile 2; brown line, tertile 3

The incidence (i.e. one minus survival probability) of cerebrovascular events of the study population in relation to the different tertiles of arteriolar tortuosity (logrank *p* = 0.003) and fractal dimension (logrank *p* = 0.015) is shown in Figs 1 and 2, respectively.

## Discussion

To our knowledge this is the first study to examine a comprehensive range of quantitative retinal traits and their relationship with incident cardiovascular events in a large longitudinal study of people with type 2 diabetes in the UK. We found that increased arteriolar tortuosity and reduced fractal dimension were associated with incident cerebrovascular events in people with type 2 diabetes over 8 years’ follow-up.

Although the association between fractal dimension and cardiovascular risk factors in the general population [20] and in people with type 1 diabetes [21] has been well documented, few studies have focused on people with type 2 diabetes with suboptimal retinal fractal dimension and cerebrovascular events. In one study, decreased arteriolar and venular fractal dimension were associated with stroke and its subtypes [22]. In a meta-analysis, narrower retinal arteriolar width and decreased fractal dimension were associated with stroke [23]. Interestingly, for these traits their direction of effect was consistent with the findings of the ET2DS analysis.

Change in fractal dimension may reflect arteriovenous differentiation after hypoxic signals during embryological development of the retinal vasculature [24]. Previous studies have found that pericyte apoptosis and activation of the renin–angiotensin system are the leading early mechanisms of the impact of diabetes on retinal microvasculature, because of the increasing activation of Angiotensin II causing impaired cell growth, angiogenesis and apoptosis, which may explain decreased vascular density [25].

In our ET2DS dataset, increased arteriolar tortuosity was also associated with incident cerebrovascular events. In a cross-sectional study, increased arteriolar and venular tortuosity were associated with ischaemic stroke in the general population [22]. Results from the Norfolk Eye Study in the UK showed that increased arteriolar tortuosity was associated with prevalent stroke, with no association with prevalent myocardial infarction [26]. Tortuous blood vessels are more common in individuals with thrombosis in internal carotid artery, atherosclerosis, hypertension, ageing and abdominal aortic aneurysm [27]. Despite, contradictions in the findings of decreased arteriolar tortuosity with hypertension and ageing [28], the direction of effect for arteriolar tortuosity in our study is consistent with previous epidemiological evidence and supports grounds for explanation of the findings.

**Fig. 3:**
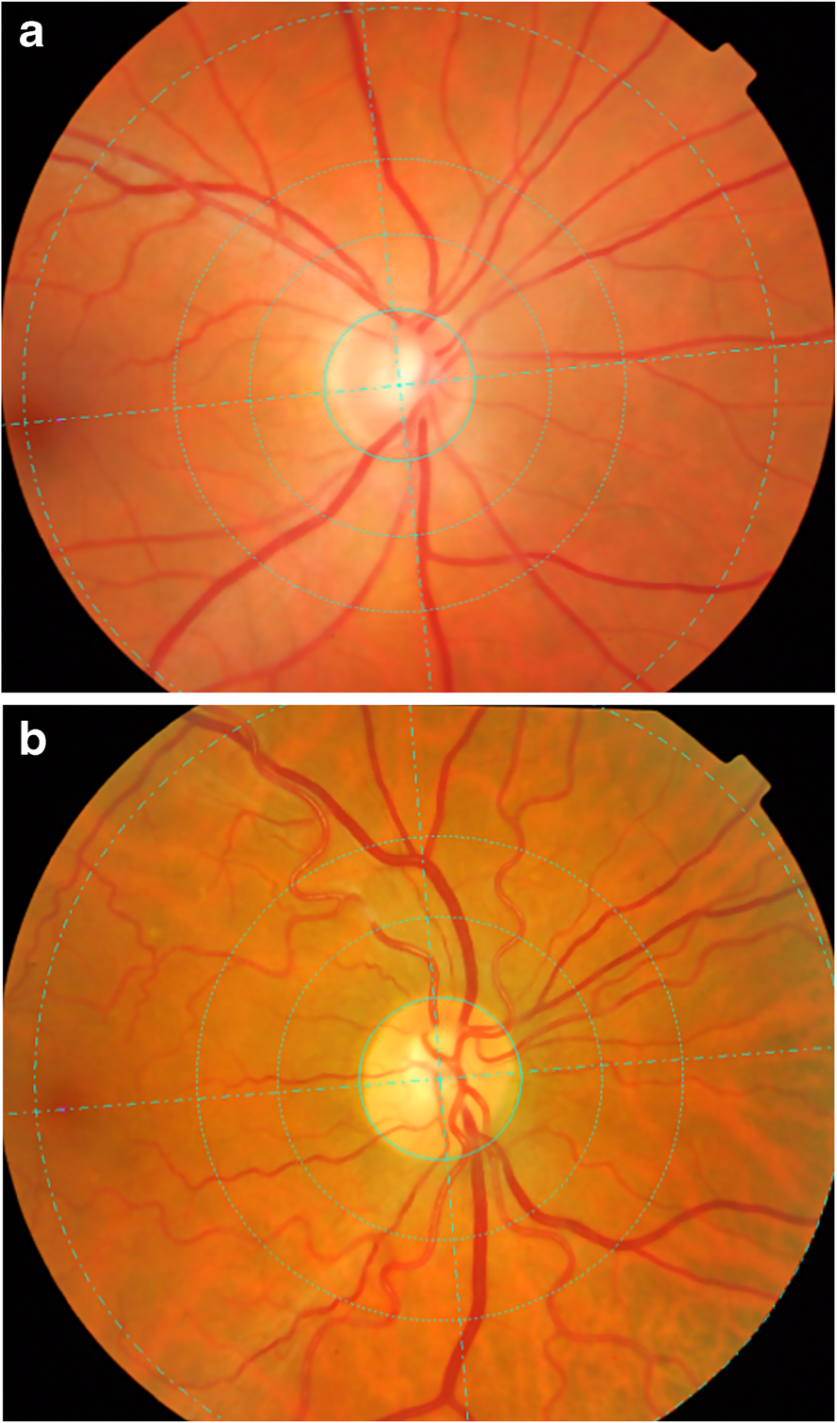
Retinal fundus photographs assessed quantitatively by VAMPIRE software. In these figures, the venules are thicker and have a darker shade of red while the arterioles are thinner with a lighter shade of red. (**a**) Image with low value of arteriolar tortuosity. (**b**) Image with high value of arteriolar tortuosity. Both images were taken from ET2DS

**Fig. 4:**
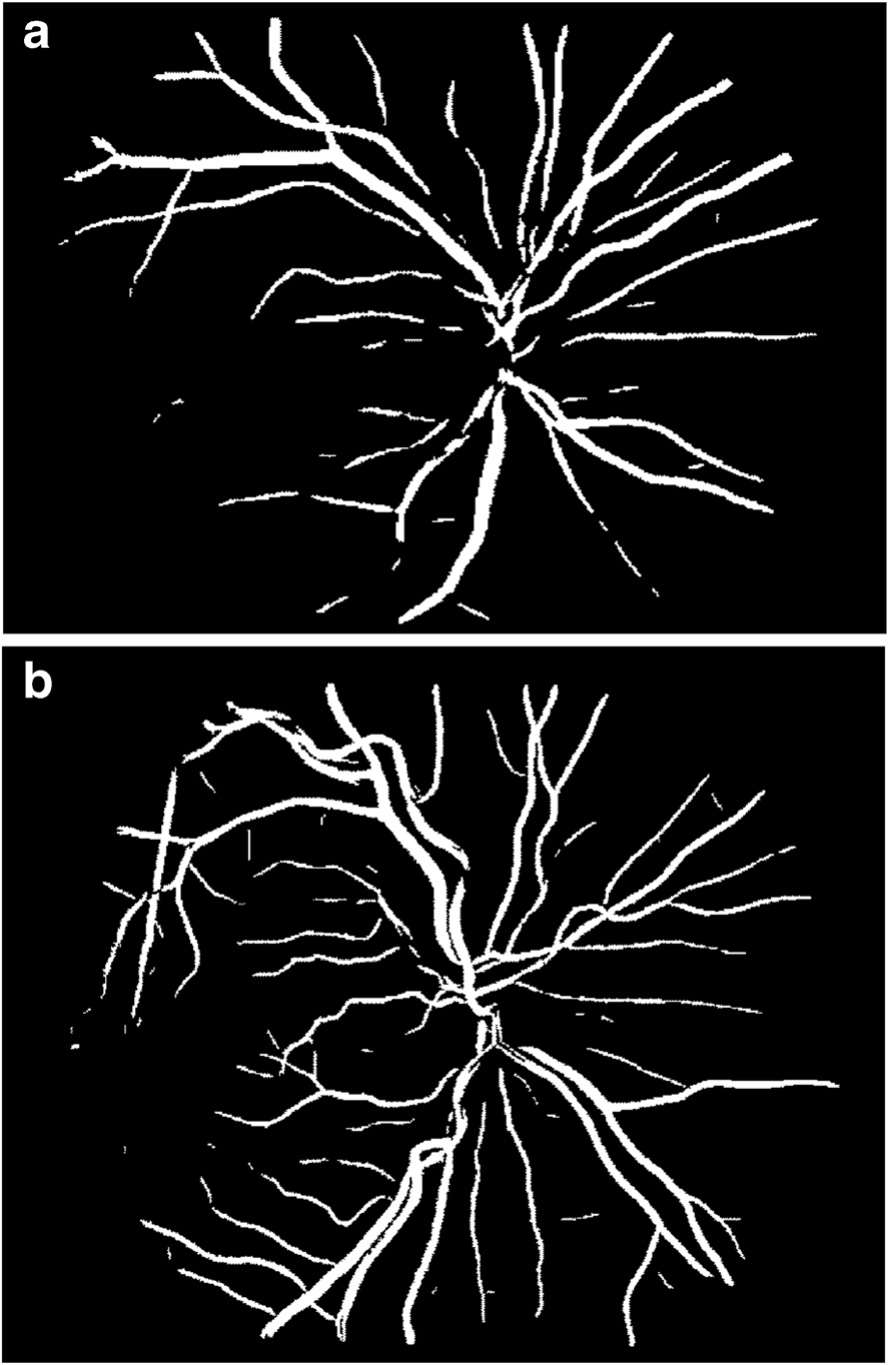
Retinal fundus photographs assessed quantitatively by VAMPIRE software. In these figures, arterioles and venules are present and were analysed from a skeletonised line tracing to obtain fractal dimension. (**a**) Image with low value of fractal dimension which indicates sparser vascular network pattern. (**b**) Image with high value of fractal dimension. Both images were taken from ET2DS

There is currently no exact pathophysiological mechanism to explain the changes seen in the retinal traits but they are likely to be associated with multiple factors including haemodynamic changes, structural changes and genetic alterations. Mechanical buckling and the loss of mechanical instability could initiate the development of arteriolar tortuosity. Tortuosity in the microvasculature might be associated with increased local stress and render atherosclerotic plaques prone to rupture [29]. Interestingly, blood dynamics studies have shown that, compared with the same sized tortuous venules, flow in the arteries generated a higher amount of mural thrombi and platelet activation rate [30] in the microvasculature, because vessel tortuosity (e.g. curvature, coils, twist and kinks) can restrict or completely occlude the blood flow which may result in stroke or CVD. Retinal microvasculature shares embryological, anatomical, and regulatory characteristics with that of the cerebral circulation [31, 32]. It is probable that retinal changes match those in the cerebral vessels, explaining the associations with stroke rather than other forms of CVD. In a study, there was no link between lacunar stroke and severe stage diabetic retinopathy, even though lacunar stroke is regarded as a microvascular cerebral disease [33]. Prospectively, the presence of severe diabetic retinopathy has been associated with incident cerebral infarction and haemorrhagic stroke [34]. The underlying explanation of our results may be that, rather than the typical signs of diabetic retinopathy (e.g. mild retinopathy), more subtle and specific changes in the retinal vascular architecture, such as changes in tortuosity and sparser vascular network pattern (Figs 3 and 4, respectively), are associated with cerebrovascular events, highlighting their importance in cardiovascular disease [35].

We did not find any evidence of association of retinal width parameters with coronary events, despite the association of narrower arterioles with incident cardiovascular events in women but not in men in the general population [10, 36]. Coronary microvascular dysfunction is frequently present in the absence of epicardial atherosclerosis (predominantly in women) [37], and non-obstructive microvascular disease is an independent risk factor for adverse cardiovascular events [10]. Once epicardial coronary disease becomes apparent, as is more often the case in higher risk men or people with type 2 diabetes, retinal vessel widths are less likely to be useful in predicting cardiovascular events.

The strengths of this study include a longitudinal analysis in a well characterised dataset with exceptionally long diabetes duration and ~8 years follow-up, enabling multivariable adjustment. This study also benefited from representativeness of type 2 diabetes population. We have a dataset with established protocol and high-quality data collection. At baseline, variables including diabetic retinopathy were characterised using routine data from high quality sources and standard operating procedures, and a systemic approach for assessing cardiovascular events, which ensured minimised loss to follow-up for cardiovascular events. High quality digital fundus images were analysed employing semi-automatic retinal vessel software VAMPIRE, which has shown reliable results in previous studies and has been useful for identifying biomarkers and association with cardiovascular risk factors and complications in people with type 2 diabetes [38]. The unique advantage of analysing retinal quantitative parameters over other biomarkers is that the retinal assessment directly reflects the microvasculature changes.

Our study also has some limitations. Replication and validation of these findings in another larger study of individuals with type 2 diabetes is warranted. It would be ideal to conduct this analysis in a prospective cohort of individuals with newly diagnosed diabetes without prevalent cardiovascular events in order to capture early vascular changes and to confirm temporal effects of the quantitative retinal parameters. The number of studies reporting quantitative retinal traits has increased greatly but there has not been homogeneity in findings, likely arising from a lack of standardisation in the algorithms and methods used across different analysis software. McGrory et al. [39] found poor agreement between software, although results with cardiovascular factors seemed application independent.

While quantitative retinal parameters could potentially be used along with other non-traditional and traditional cardiovascular risk factors for the stratification of cerebrovascular risk, the increment in C statistic, which we found when retinal parameters were added to a model already containing a range of traditional risk factors, was relatively small. Although a modest increase in C statistic is consistent with findings from other studies on vascular risk prediction and may be related to the insensitivity of the C statistic when used in this context [40], our findings do not support immediate incorporation of the parameters into risk prediction scores. Further investigation on the potential clinical utility of these parameters, including better understanding of vascular aetiologies and measurement in clinical trials, may improve predictive ability.

## Supplementary Information

ESM (PDF 122 kb)

## Data Availability

The summary data that support the findings of this study are available from the corresponding author on reasonable request.

## Abbreviations

AVR: Arteriovenous ratio
CRAE: Central retinal arteriolar equivalent
CRP: C-reactive protein
CRVE: Central retinal venular equivalent
ET2DS: Edinburgh Type 2 Diabetes Study
GP: General practitioner
ISD: Information service division
TIA: Transient ischaemic attack
VAMPIRE: Vascular Assessment and Measurement Platform for Images of the Retina
