# Supplementary material for: Retinal arteriolar tortuosity and fractal dimension are associated with long-term cardiovascular outcomes in people with type 2 diabetes
Source: Diabetologia. 2021 Jun 23;64(10):2215–27. doi: 10.1007/s00125-021-05499-z (PMC8423701; doi:10.1007/s00125-021-05499-z)
Supplement: Supplementary file 1 — (PDF 122 kb) [file 125_2021_5499_MOESM1_ESM.pdf]

Electronic Supplementary Material (ESM)

ESM Table 1. Association of quantitative retinal traits with incident severe cardiovascular events (myocardial infarction, coronary intervention, fatal ischaemic heart disease and stroke) in Cox regression analyses.

ESM Fig. 1. Measurement of retinal vascular traits (e.g. arterioles and venules diameters) from a retinal photograph using VAMPIRE.

•

		AVR			CRAE			CRVE		
	No. of Events	HR	95% CI	*p* value	HR	95% CI	*p* value	HR	95% CI	*p* value
**Severe CV event**	152									
Model A		0.38	0.05-2.96	0.36	0.98	0.93-1.01	0.29	0.99	0.96-1.02	0.80
Model B		0.37	0.05-2.75	0.33	0.98	0.94-1.03	0.58	1.00	0.97-1.03	0.78
Model C		0.47	0.06-3.55	0.46	0.96	0.93-1.02	0.14	0.98	0.95-1.01	0.36
	152	**Arteriolar tortuosity**			**Venular tortuosity**			**Fractal dimensions**		
		HR	95% CI	*p* value	HR	95% CI	*p* value	HR	95% CI	*p* value
Model A		1.05	0.91-1.20	0.49	1.00	0.83-1.20	0.94	0.87	0.74-1.01	0.08
Model B		1.06	0.91-1.21	0.45	1.00	0.83-1.20	0.97	0.88	0.75-1.03	0.13
Model C		1.03	0.89-1.20	0.63	0.94	0.77-1.14	0.54	0.86	0.72-1.02	0.08

ESM Table 1. Association of quantitative retinal traits with incident severe cardiovascular events (myocardial infarction, coronary intervention, fatal ischaemic heart disease and stroke) in Cox regression analyses. Model A: unadjusted. Model B: Basic model adjusted for sex and age. Model C: multivariate, fully adjusted for CV risk factors (sex, age, duration of diabetes, systolic blood pressure, HbA_{1c},, total cholesterol, HDL cholesterol, smoking, prevalent baseline cardiovascular disease, estimated glomerular filtration rate and diabetic retinopathy). AVR, arteriovenous ratio; CRAE, central retinal arteriolar equivalent; CRVE, central retinal venular equivalent

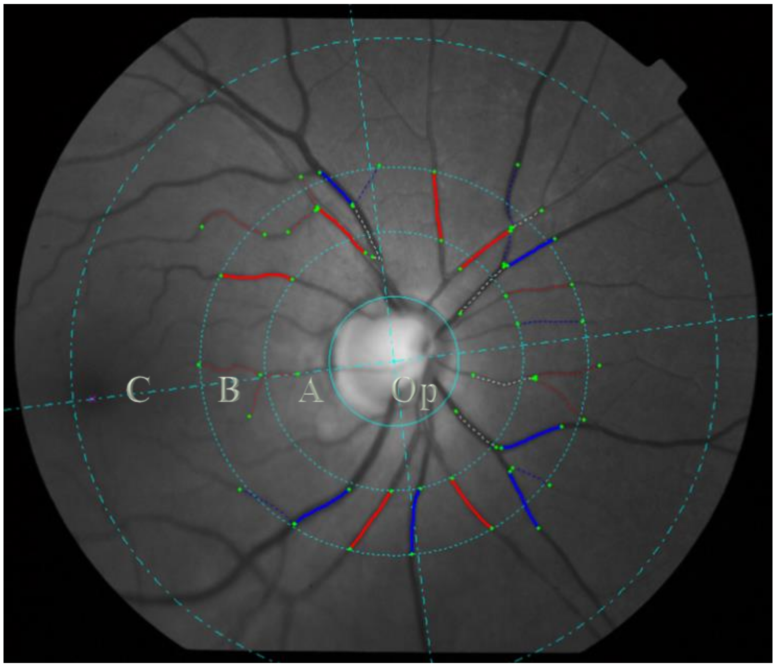

ESM Fig. 1. Measurement of retinal vascular traits (e.g. arterioles and venules diameters) from a retinal photograph using VAMPIRE. This software automatically identifies the optic disc, places a grid with reference to its centre, identifies vessel type and calculates retinal measurements. At the centre, is located the optic disc (Op) and zone A(A) is next to this area. CRAE and CRVE are measured from zone B (B). The adjacent area, zone C (C), is where arteriolar and venular tortuosity are measured. The arterioles are highlighted in red and the venules are highlighted in blue. Measurement of retinal variables is performed by trained graders who are responsible for the visual evaluation of VAMPIRE's semi-automated measurements and performing manual intervention if necessary. CRAE, Central Retinal Arteriolar Equivalent; CRVE, Central Retinal Venular Equivalent.
